# Combined impact of gray and superficial white matter abnormalities: Implications for epilepsy surgery

**DOI:** 10.1111/epi.18494

**Published:** 2025-06-10

**Authors:** Csaba Kozma, Jonathan Horsley, Gerard Hall, Callum Simpson, Jane de Tisi, Anna Miserocchi, Andrew W. McEvoy, Sjoerd B. Vos, Gavin P. Winston, Yujiang Wang, John S. Duncan, Peter N. Taylor

**Affiliations:** ^1^ CNNP Lab (www.cnnp‐lab.com), School of Computing Newcastle University Newcastle upon Tyne UK; ^2^ Department of Clinical and Experimental Epilepsy, UCL Queen Square Institute of Neurology University College London London UK; ^3^ Western Australia National Imaging Facility The University of Western Australia Nedlands Australia; ^4^ Centre for Medical Image Computing, Computer Science Department University College London London UK; ^5^ Department of Medicine Queen's University Kingston Ontario Canada; ^6^ Faculty of Medical Sciences, Translational and Clinical Research Institute Newcastle University Newcastle upon Tyne UK

**Keywords:** epilepsy surgery, gray matter, multimodal, superficial white matter

## Abstract

**Objective:**

Drug‐resistant focal epilepsy is associated with abnormalities in the brain in both gray matter (GM) and superficial white matter (SWM). However, it is unknown if both types of abnormalities are important in supporting seizures. Here, we test if surgical removal of GM and/or SWM abnormalities relates to post‐surgical seizure outcome in people with temporal lobe epilepsy (TLE).

**Methods:**

We analyzed structural imaging data from 143 patients with TLE (pre‐op diffusion magnetic resonance imaging and pre‐op T1‐weighted MRI) and 97 healthy controls. We calculated GM volume abnormalities and SWM mean diffusivity abnormalities and evaluated if their surgical removal distinguished seizure outcome groups post‐surgically.

**Results:**

At a group level, GM and SWM abnormalities were most common in the ipsilateral temporal lobe and hippocampus in people with TLE. Analyzing both modalities together, compared to in isolation, improved surgical outcome discrimination (GM area under the curve [AUC] = 0.68, *p* < 0.01; WM AUC = 0.65, *p* < 0.01; Union AUC = 0.72, *p* < 0.01; Concordance AUC = 0.64, *p* = 0.04). In addition, 100% of people who had all concordant abnormal regions resected had International League Against Epilepsy (ILAE)_1,2_ outcomes.

**Significance:**

Resecting abnormalities in GM or SWM individually affects surgical outcomes but combining both provides clearer patient group distinctions. This approach improves outcome differentiation, showing higher rates of patients living without disabling seizures when all concordant abnormal regions are resected. These findings suggest that regions identified as abnormal from both diffusion‐weighted and T1‐weighted MRI are involved in the epileptogenic network and that resection of both types of abnormalities may enhance the chances of living without disabling seizures.

**Key points**

- Abnormalities in both gray matter (GM) and superficial white matter (SWM) are found in patients with epilepsy, helping inform surgical decisions.
- Incorporating both GM and SWM together improves surgical outcome differentiation.
- Removing concordant abnormal regions further increases the rate of living without disabling seizures.

## 1 INTRODUCTION

Approximately half of the people who undergo epilepsy surgery still experience seizures postoperatively, likely due to incomplete resection of the epileptogenic zone.^1^ Successful outcomes depend on the precise localization and removal or disconnection of seizure‐generating tissue.^2^, ^3^ Although gray matter (GM) and superficial white matter (SWM) abnormalities are often assessed separately, they may contribute jointly to the epileptogenic network. This study examines the relationship between resection of both GM and SWM abnormalities and post‐surgical outcomes in individuals with temporal lobe epilepsy (TLE).

People with TLE commonly have GM atrophy in the temporal, frontal, and parietal cortices, along with subcortical structures, including the thalamus and amygdala, and damage to WM tracts, especially in the limbic and subcorticocortical regions, particularly close to the epileptogenic zone.^4^, ^5^, ^6^, ^7^ GM atrophy in TLE often follows a widespread, multi‐lobar, bilateral pattern.^4^ WM damage is lateralized predominately to the hemisphere of seizure focus.^4^, ^8^ Hippocampal sclerosis (HS) is associated with both SWM damage and extensive cortical thinning.^6^, ^9^, ^10^ SWM abnormalities can complement T1‐weighted (T1w) imaging by revealing epileptogenic zone features invisible on T1‐weighted alone.^6^, ^11^ Because SWM potentially reflects local connectivity, including ‘u” fibers, its contribution is essential for a more detailed understanding of epileptogenic activity.^12^ Few studies have specifically investigated the connection between GM and WM abnormalities,^13^, ^14^, ^15^ and cortical thinning and damage to WM may result from separate underlying processes.^6^, ^13^

Resection of structurally abnormal regions is associated with an increased chance of seizure freedom.^8^, ^16^, ^17^, ^18^, ^19^ Furthermore, removing nodes or tracts with abnormal diffusion may also improve outcomes.^20^, ^21^, ^22^, ^23^ SWM improves tissue characterization by detecting subtle abnormalities and precisely localizing epileptic regions, guiding surgical decisions to optimize patient outcomes.^20^ Although each approach may aid in identifying epileptogenic tissue, few studies have examined GM and WM abnormalities together in the context of epilepsy surgery.^6^, ^24^ Our study aims to fill this gap and emphasize the advantages of jointly analyzing GM and SWM abnormalities in this context.

This study examines the relationship between the resection of abnormal regions in GM and SWM and post‐surgical outcomes in 143 individuals with TLE who underwent resective surgery. We identified regions characterized by reduced GM volume and increased SWM mean diffusivity (MD) and compared these regions to individual resection masks. This allowed us to evaluate how effectively each modality—individually and in combination—differentiates between favorable outcomes (International League Against Epilepsy [ILAE]_1,2_) and less favorable ones (ILAE_3+_).

## 2 METHODS

### 2.1 Subjects

We conducted a retrospective study of 143 individuals with TLE at the National Hospital for Neurology and Neurosurgery, London, who had temporal lobe resections between 2009 and 2021. This study of anonymized data that had been acquired previously was approved by the Health Research Authority, without the need to obtain consent from each subject (University College London Hospital epilepsy surgery database: 22/SC/0016), and by the Database Local Data Monitoring Committee. Individuals who declined for their data to be used in anonymized research were not included in the research database. Patients were matched with 97 healthy controls, who provided individual written consent (Table 1), and data were collected using two acquisition protocols. All participants underwent anatomic T1‐weighted and diffusion‐weighted magnetic resonance imaging (MRI), with TLE laterality assessed through pre‐surgical evaluations. Age at onset of epilepsy ranged from 1 to 52 years (median = 15 years, interquartile range [IQR] = 15.75 years), and 53% of patients had HS (Table 1). Postoperatively, 73.4% achieved ILAE_1,2_ seizure outcomes at 12 months (Table 1). Results regarding ILAE outcomes for Years 2–5 are provided in the Data S1.

**TABLE 1:** Control and patient data by outcome at 1 year.

	Control	$\mathrm{ILA}E_{1,2}$	$\mathrm{ILA}E_{3+}$	Test statistic
*N*	97	105	38	
Age at onset, median (IQR)	–	15 (15)	17.5 (11.8)	W=2381,p=0.08
Age at scan, median (IQR)	39 (20.8)	36.9 (17.3)	41.7 (15.4)	K=2.409,p=0.30
Sex, male:female	37:60	43:62	19:19	$X^{2}=1.32,p=0.52$
Side, left:right	–	62:43	19:19	$X^{2}=0.60,p=0.43$
HS, Yes:No	–	60:45	16:22	$X^{2}=9.49,p=0.15$

### 2.2 Data acquisition

Individuals were scanned using one of two acquisitions. The first cohort (87 patients, 29 controls) was scanned between 2009 and 2013, using a 3T GE Signa HDx scanner with standard imaging gradients (40 mTm‐1, 150 Tm‐1s‐1) and an 8‐channel phased array coil. T1‐weighted images were acquired with a three‐dimensional (3D) inversion‐recovery fast spoiled gradient recalled echo (FSPGR) sequence (Repetition time (TE) = 3.04 ms, Echo time (TR) = 37.68 s, 170 contiguous 1.1 mm coronal slices each a 256 × 256 matrix with 0.9375 × 0.9375 mm in plane resolution). Diffusion‐weighted images were collected with a cardiac‐triggered single shot spin‐echo planar imaging (EPI) sequence (TE = 73 ms) with 60 axial slices. Each slice was 2.4 mm in size with a 96 × 96 matrix, zero‐filled to 128 × 128, with 1.875 × 1.875 mm in‐plane resolution. Overall 52 diffusion directions (*b* = 1200 s/mm^2^ [δ = 21 ms, Δ = 29 ms, maximum gradient strength]) with 6 B0 scans were collected.

The second cohort (56 patients, 68 controls) was scanned between 2014 and 2019, using a 3T GE MR750 scanner with a higher gradient strength (50 mTm‐1, 200 Tm‐1s‐1) and a 32‐channel phased array coil. T1‐weighted images were collected with an FSPGR sequence (TE = 3.1 ms, TR = 7.4 ms, TI = 400 ms, 170 contiguous 1 mm coronal slices each 256 × 256 with 1 mm × 1 mm in plane resolution). Diffusion‐weighted MRI used a cardiac‐triggered single‐shot EPI sequence (TE = 74.1 ms) with 70 axial slices and 115 volumes across four *b*‐values (0, 300, 700, 2500 s/mm^2^ [δ = 21.5 ms, Δ = 35.9 ms]). Overall, 11 B0 images were collected, with a field of view of 256 × 256 mm and an acquisition matrix size was 128 × 128. The final reconstructed voxel size was 2 × 2 × 2 mm.

### 2.3 Data processing and registration

The Diffusion Magnetic Resonance Imaging scans from both cohorts underwent identical data processing, including de‐noising,^25^ Gibbs‐unringing,^26^ and signal drift correction.^27^ Because one cohort lacked reverse phase‐encoded B0s, the Synb0‐DisCo tool^28^, ^29^ generated non‐distorted synthetic images from T1 structural diffusion MRI for all participants. This tool was applied to both cohorts to maintain consistency. The synthetic images were then signal bias corrected using N4,^30^ and processed with TOPUP^31^, ^32^ and EDDY to address warping,^33^ and eddy current distortions, and motion. After pre‐processing, tensor maps were calculated via FSL's DTIFIT tool.^32^ MD maps were computed directly registered to standard space using linear (affine) and non‐linear (SYN‐Diffeomorphic) registrations using the ANTs toolbox.^34^ All MD maps were registered to the “FMRIB158_2mm” MD template provided from the FSL toolbox.^32^ We investigated MD maps, as these were shown recently to hold potentially valuable localizing information.^20^ Once registered, both linear and non‐linear transformations were applied to all tensor maps with a trilinear interpolation. To examine SWM, only WM voxels within 5 mm of the GM–WM boundary in the selected regions of interest (ROIs) were included in the analysis. A threshold of 5 mm was chosen based on previous work demonstrating this as a limit for the largest difference to controls.^12^

T1‐weighted MRI studies were used to generate parcellated GM ROI. FmreeSurfer's “recon‐all” pipeline^35^ was applied for intensity normalization, skull stripping, subcortical volume generation, and parcellation, following ENIGMA guidelines. We used the most detailed version of the Lausanne parcellation,^36^ with 446 neocortical and 14 deep brain regions, including the hippocampus, amygdala, thalamus, putamen, and caudate. All FreeSurfer‐generated surfaces were inspected visually for accuracy and corrected where necessary as described previously.^37^

### 2.4 Abnormality calculation

**FIGURE 1:**
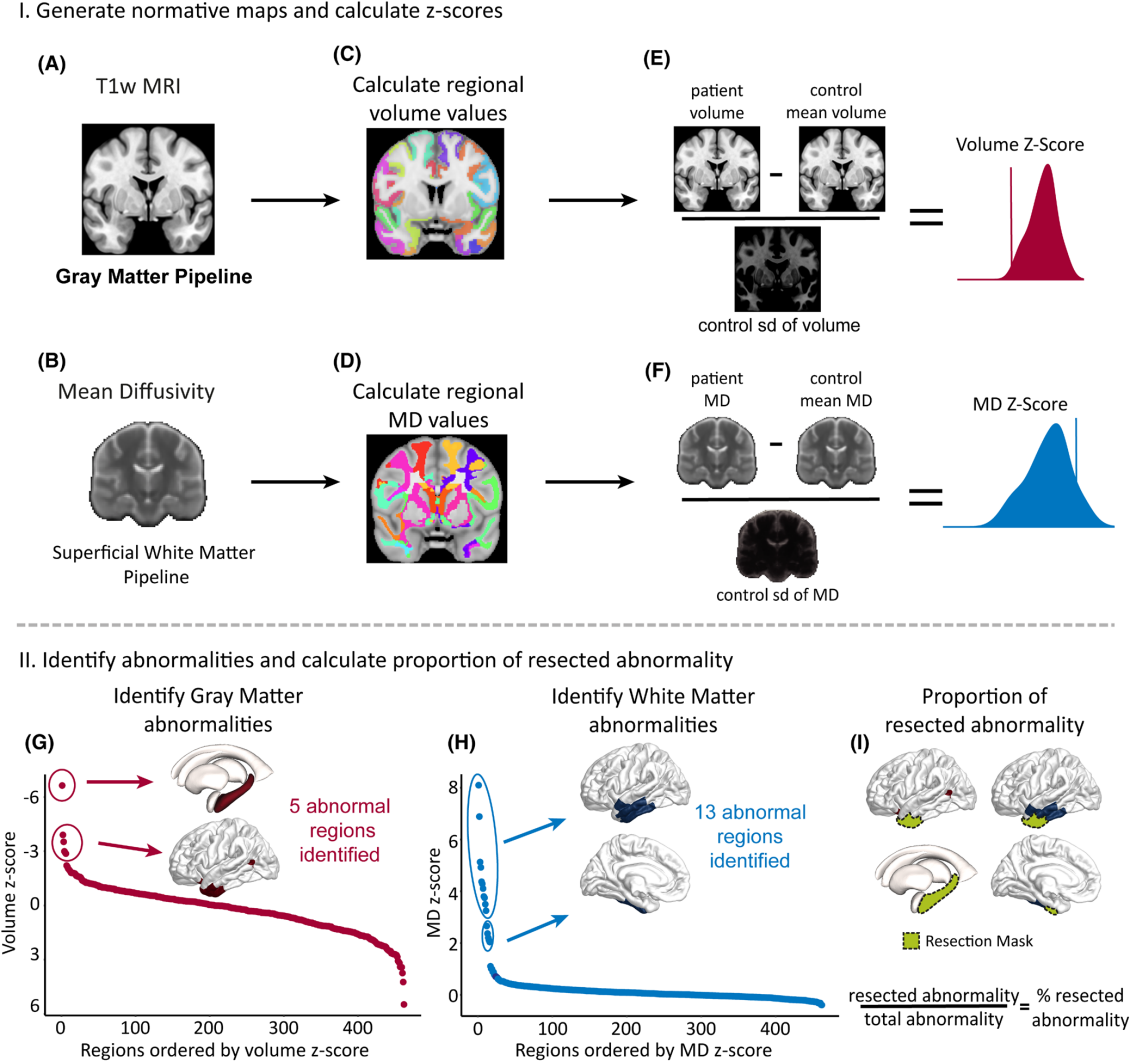
I. Generate normative maps and calculate *z*‐scores. (A, B) Individual T1‐weighted magnetic resonance imaging (MRI) and mean diffusivity (MD) maps provide volume and superficial white matter MD values. (C, D) Regional volume and MD values are calculated and then (E, F) *z*‐scored against the normative map to indicate deviations from the healthy mean. II. Identify abnormalities and calculate proportion of resected abnormality. (G, H) *Z*‐scores are ranked per modality, and change point analysis identifies abnormal regions (regions of interest [ROIs]). (I) For each subject, abnormality distributions are overlaid with the resection mask to calculate the proportion of resected abnormal ROIs.

We used the healthy controls to compute normative baselines of GM volume and adjacent SWM MD per region. We selected MD because it reflects average diffusion in all directions, with higher MD values indicating more unrestricted diffusion, often linked to myelin disruption and increased extracellular space in WM,^38^, ^39^ which are common in epilepsy.^7^ For the SWM, we took the MD of white matter voxels within 5 mm of the nearest region in the Lausanne parcellation. We calculated regional means and standard deviations (SDs), harmonizing across scanning protocols using ComBat^40^ and adjusting for covariates (age and sex). For individuals with TLE, we calculated abnormalities by *z*‐scoring each region's MD and volume values against the corresponding normative map. These abnormalities quantified deviations from the healthy mean in each region for both modalities (Figure 1A–F). We focused on negative *z*‐scores for GM abnormalities, reflecting volume reductions. For SWM, we analyzed positive *z*‐scores in MD, which indicate increased diffusion.^5^, ^7^

Within each patient, regional abnormalities were ranked in each modality separately. The ranking was set from the most negative to the most positive for GM (we expect volume loss to be abnormal) and the opposite for SWM (we expect MD increase as abnormal). Then, multiple change point (MCP) analysis was applied to identify patient‐specific thresholds to find abnormal ROIs (Figure 1G,H).^41^ MCP analysis uses Bayesian regression to locate shifts in means, variances, or autocorrelation^41^; here, it identified mean shifts as thresholds for abnormal ROIs compared to the rest of the distribution of regions. We examined the spatial distribution of regional abnormalities in GM, SWM, their union (GM, SWM, or both), and the concordance of GM and SWM. We have included these terms and their definitions in Table S11.

### 2.5 Resection mask generation

Resection masks were generated using a semi‐automated method.^42^ Postoperative imaging was used to create masks of the resected tissue in preoperative space. This process began with an automated pipeline incorporating FastSurfer,^43^ ANTs,^34^ and ATROPOS^44^ to produce the initial masks. These masks were then visually inspected for accuracy and manually corrected if necessary.^37^ Most patients (~93%) underwent the same surgical approach with larger resections in the non–language‐dominant (typically right) hemisphere, whereas ~7% underwent lesionectomies. Once finalized, the resection masks were aligned to the Montreal Neurological Institute (MNI)‐152 standard space, matching the space of the abnormality maps. Regions with more than 10% overlap with the resection mask were considered as resected. We present the overall average resection overlap across the whole cohort in Figure S12.

The vast majority (>80%) of postsurgical scans were acquired within 12 months of surgery using a 3D T1 sequence at the National Society for Epilepsy (Chalfont St Peter, UK). However, for some patients where this was unavailable, scans from the National Hospital for Neurology & Neurosurgery (NHNN) were used instead. Although most scans were acquired at Chalfont, 7 of the 143 scans were from NHNN. For NHNN scans, acquisition parameters varied, as did the date of acquisition relative to surgery. Particular attention was paid to post‐operative brain shift and sagging into the resection cavity, especially for scans acquired >3 months postoperatively. The median interval between resection surgery and post‐operative T1‐weighted MRI acquisition was 5.76 months (range: 0.61–96.85 months) in the Chalfont cohort and 5.27 months (range: 0.01–14.37 months) in the NHNN cohort. Further details on resection masks and their generation can be found in our previous work.^37^

For each subject, the abnormality distributions were overlaid with the resection mask to compare the spatial overlap of resected tissue with abnormal ROIs (Figure 1I). We calculated the proportion of abnormal ROIs that had been resected and investigated whether this differed between ILAE_1,2_ and ILAE_3+_ cases.

### 2.6 Statistical analysis

After computing the proportion of abnormalities resected in GM, SWM, or both, we compared these values between groups that were subsequently free of disabling seizures (ILAE_1,2_) or not (ILAE_3+_). To measure the magnitude of difference between groups we measured the area under the receiver‐operating characteristic (ROC) curve (AUC). To assess its significance, we used a one‐tailed Kruskal–Wallis test, as we had a prior hypothesis that patients without disabling seizures would have a greater proportion of their abnormalities removed. To further evaluate the added value of using the combined contribution of multiple modalities (GM and SWM), we conducted individual binomial logistic regression analyses using proportions of resected abnormalities including only GM, only SWM, or their union, and then compared the models using a likelihood ratio test.

## 3 RESULTS

### 3.1 Abnormality is present in gray matter and superficial white matter

**FIGURE 2:**
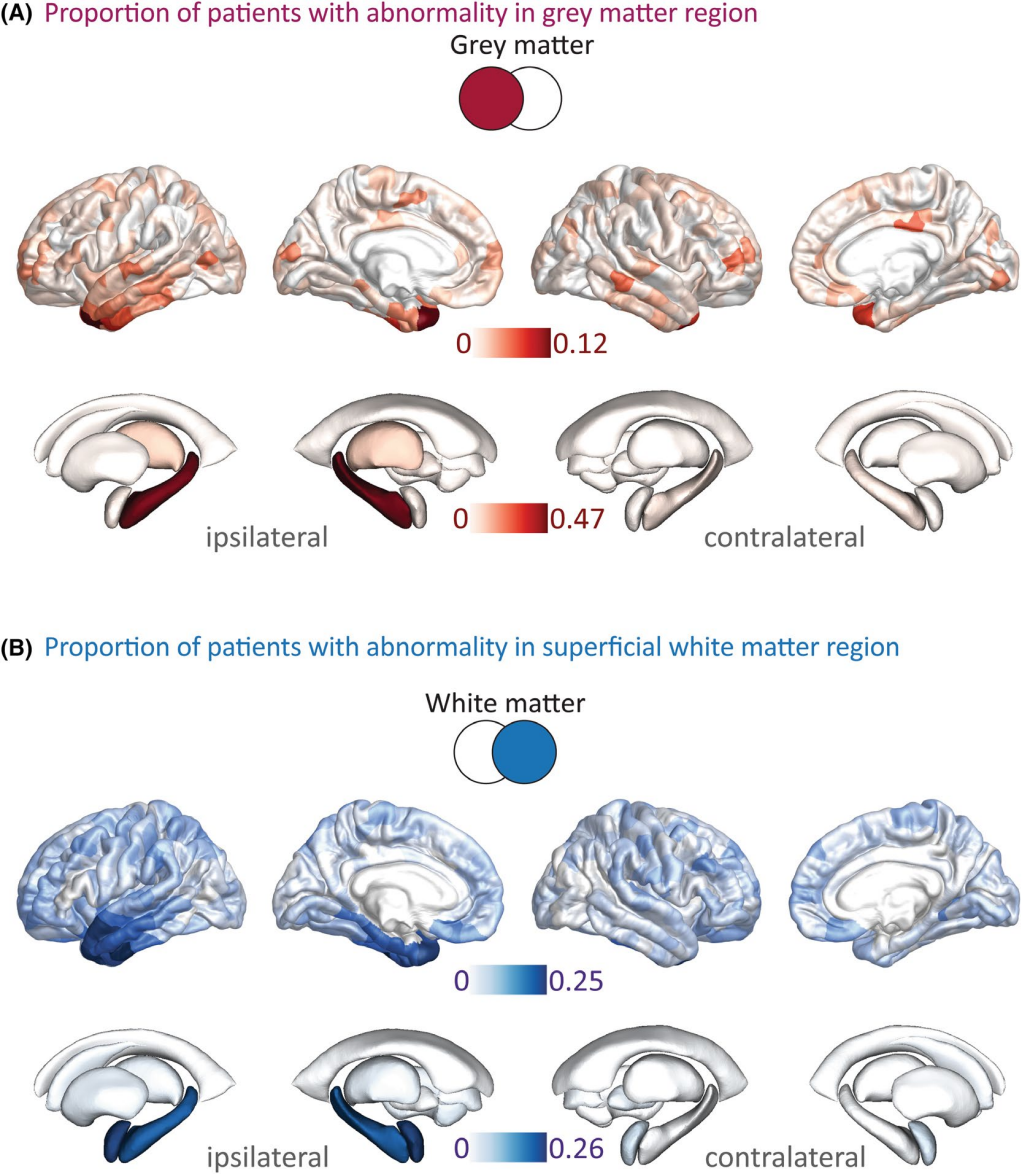
Spatial distribution of gray matter and superficial white matter abnormalities. The proportion of individuals with abnormalities in each region of interest for (A) gray matter and (B) superficial white matter.

Abnormalities in both GM and SWM were present across the whole cohort (Figure 2). GM atrophy in neocortical regions was seen in a widespread, bilateral pattern affecting the temporal, frontal, and parietal lobes. The greatest proportion of abnormalities were in the ipsilateral hippocampus (47%), and ipsilateral temporal pole, where 12% of patients showing abnormally reduced volume. (Figure 2A). SWM abnormalities were more discrete, primarily ipsilateral and near to the epileptogenic zone. Twenty‐six percent had abnormal MD adjacent to the ipsilateral hippocampus, and 25% of patients had abnormal ipsilateral inferior temporal gyrus MD (Figure 2B).

### 3.2 Resection of gray matter or superficial white matter abnormalities relates to post‐surgical seizure outcome

**FIGURE 3:**
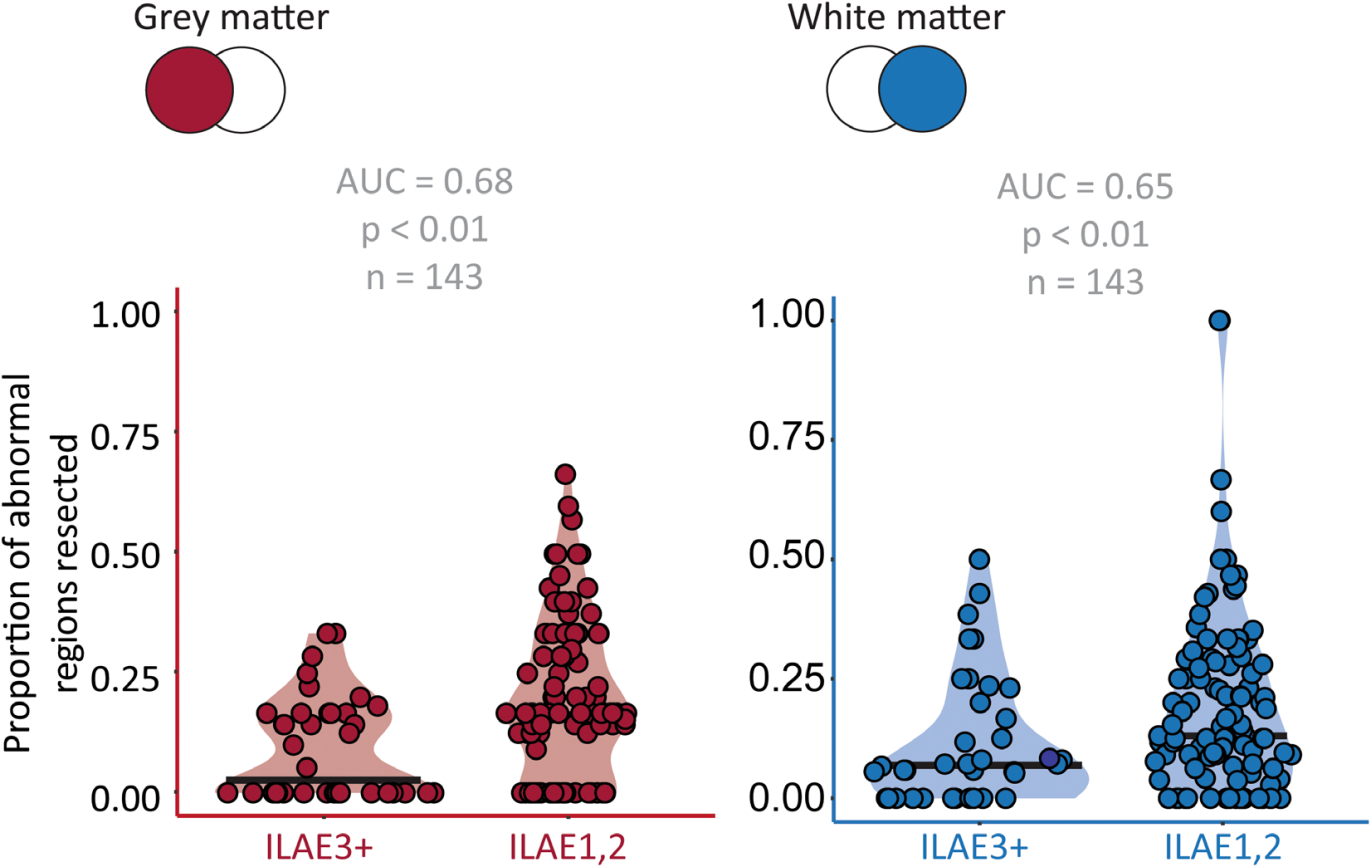
Resecting gray matter (GM) and superficial white matter (SWM) abnormalities differentiates post‐surgical outcome. The proportion of abnormal regions resected distinguished post‐surgical outcome using both (A) GM and (B) SWM. Each data point represents an individual patient. The darker line shows the median. Areas under the curve were derived from receiver‐operating characteristic curves using the resected regions of interest to differentiate surgical outcomes.

Next, abnormality distributions were overlaid with resection masks to calculate the proportion of resected abnormal ROIs and compare the ILAE_1,2_ and ILAE_3+_ cases. Resecting abnormal GM ROIs significantly differentiated patient outcomes (AUC = 0.68, *p* < 0.01; Figure 3A). Similarly, resecting abnormal SWM ROIs significantly differentiated patient outcomes (AUC = 0.65, *p* < 0.01; Figure 3B). Thus, when considered separately, the resection of GM or SWM abnormalities explains post‐surgical seizure outcome moderately well.

### 3.3 Combining gray matter and superficial white matter abnormalities improves outcome distinction

**FIGURE 4:**
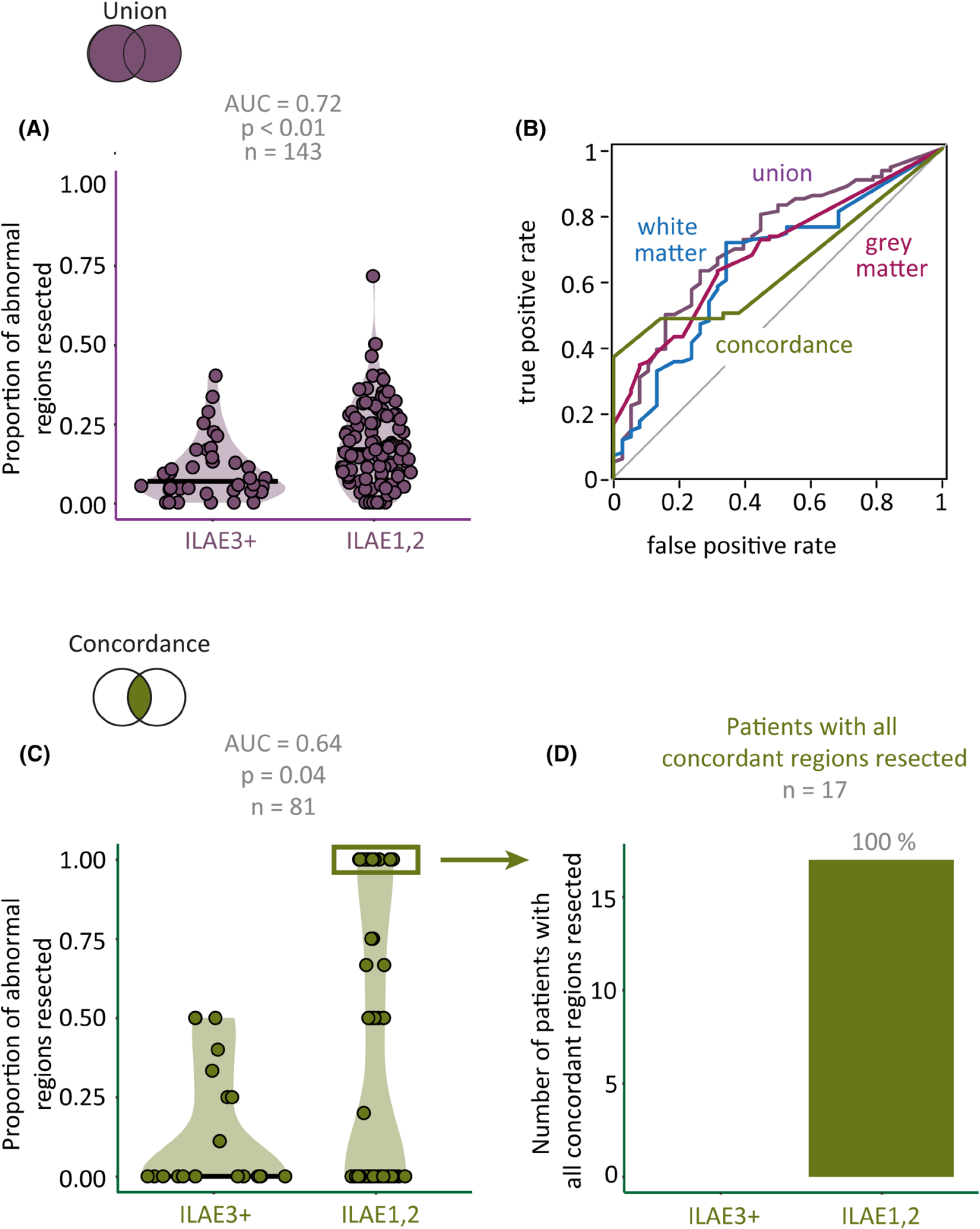
Resecting abnormal regions in gray matter (GM) and superficial white matter (SWM; union) improves the prediction of post‐surgical seizure outcome. All patients with complete resection of concordant abnormal regions of interest (ROIs) achieved International League Against Epilepsy (ILAE)_1,2_ outcomes. (A) Proportion of resected abnormal regions (GM and SWM union) distinguishes patients without disabling seizures. Each point represents a patient; the darker line shows the median. (B) Receiver‐operating characteristic curve comparing GM, SWM, their union, and concordance in predicting surgical outcomes. (C) Resected abnormal ROIs compared between ILAE_1,2_ and ILAE_3+_ patients using GM and SWM concordance. Each point represents a patient; the darker line shows the median. (D) Surgical outcomes for patients with all concordant ROIs resected.

We investigated whether resection of ROIs containing abnormalities is associated with a greater chance of freedom from disabling seizures, irrespective of their GM or SWM origin (Figure 4A,B). The surgical resection of ROIs containing GM, or SWM, or both, better differentiated seizure outcome compared to each of the modalities in isolation (AUC = 0.72, *p* < 0.01; Figure 4A). This result suggests that both modalities separately and together are indicated in abnormal activity. In addition, the logistic regression analysis indicated that the results showed that using the union of GM and SWM proportions of resected abnormalities better differentiated outcome groups than using GM or SWM only (GM vs Union: *X* ^2^ = 1.492, *p* < 0.001; SWM vs Union: *X* ^2^ = 8.456, *p* < 0.001).

Results showed that concordant abnormalities (GM and SWM overlap) were present in 56% of cases but moderately predicted post‐surgical seizure outcome (AUC = 0.64, *p* = 0.04; Figure 4C). Notably, all patients with complete resection of abnormal concordant ROIs (most frequently the ipsilateral hippocampus) had ILAE_1,2_ outcomes (Figure 4C,D).

For completeness, we present discordant GM and SWM abnormalities (Figure S1), which differentiate patient outcomes as well as their union—unsurprising because most union abnormalities are discordant. This supports the value of multimodal analysis. We also show the spatial distribution of union, concordant, and discordant abnormalities (Figure S2). Results remained consistent across acquisition protocols (Figure S4), HS and non‐HS cases (Figure S6), and MRI negative cases (Figure S3). Coarser Lausanne parcellations (216 + 14 and 114 + 14 regions) confirmed consistent outcome predictions (Figure S5), and surgical outcome findings were replicated across ILAE Year 2–5 outcomes (Figure S7). GM and SWM z‐score correlations showed no significant difference between patients and controls (*W* = 7119, *P* = 0.6592; Figure S8). Bootstrapping analysis with 1000 resamples showed consistent AUCs across the data sets, confirming the reliability of the model (Figure S9). Finally, in Table S10, we present the average proportions of regions identified as abnormal and the average proportions of abnormal regions resected across the patient cohort for GM, WM, the union, concordance, and discordance of GM and WM.

## 4 DISCUSSION

This study combined GM and SWM abnormalities to identify epileptogenic tissue in TLE in the context of epilepsy surgery. Looking at each modality in isolation, we found that the resection of GM or SWM abnormalities was clearly associated with outcome. By combining the two modalities together to include all abnormality, we found that group differentiation improved substantially. Finally, although concordance was present in only 56% of patients, complete resection consistently led to ILAE_1,2_ outcomes.

This study advances preoperative imaging research for distinguishing TLE surgical outcomes by examining combined GM and SWM abnormalities, which previous studies typically analyzed separately. Removing GM regions with abnormal volume, particularly in the hippocampus and entorhinal cortex, may be beneficial.^8^, ^16^, ^17^, ^18^ We observed GM atrophy in the ipsilateral hippocampus,^5^, ^17^, ^45^ extending bilaterally into temporal, frontal, and centroparietal areas.^4^, ^8^ Diffuse GM abnormalities, noted by,^5^, ^46^ aid in differentiating outcomes, with frontal GM atrophy and hippocampal atrophy or resection extent as reliable indicators.^17^, ^47^ Our results indicate that GM abnormalities alone more effectively distinguish ILAE_1,2_ from ILAE_3+_ outcomes in HS cases than in non‐HS cases, underscoring the importance of hippocampal volumetry in clinical evaluation. SWM abnormalities, concentrated ipsilaterally near the epileptogenic zone,^7^, ^48^, ^49^ also correlate with HS, which associates with cortical thinning.^6^, ^9^, ^10^ In TLE, resecting the temporal pole (including the piriform cortex), hippocampus, and adjacent WM is critical.^50^, ^51^ Advances in hippocampal mapping show that posterior hippocampal resection is often unnecessary.^51^ Regions such as the ipsilateral hippocampus, parahippocampal gyri, amygdala, and adjacent temporal WM structures show consistent GM/WM abnormalities across studies.^5^, ^6^ These mesiotemporal abnormalities often extend to the frontal lobes, thalamus, and cingulate cortex, mirroring the diffuse GM atrophy and SWM changes noted in our results. In addition, anterior temporal lobe resection offers a higher liklihood of seizure freedom compared to lesionectomy^1^, ^20^ found that resecting large SWM abnormality clusters significantly improves seizure freedom. Our findings suggest that targeting both GM and SWM abnormalities together may enhance the likelihood of living without disabling seizures compared to addressing these modalities separately.

We found that combining GM and SWM abnormalities, considering both their union and discordant contributions, substantially improves surgical outcome differentiations over assessing each in isolation. This suggests that distinct biological processes might drive these two types of injury in TLE, as discussed in prior studies.^6^, ^13^, ^14^, ^15^, ^46^ The coexistence of GM volume loss and SWM abnormalities in regions such as the temporal pole and frontal SWM underscores their combined role in surgical outcomes, where resection of concordant GM–SWM regions (e.g., anterior hippocampus and adjacent WM) improves the rate of living without disabling seizures. However, our finding that GM abnormalities better predict outcomes in HS cases aligns with prior studies,^5^, ^17^, ^46^ showing HS‐associated cortical thinning. Although GM–SWM concordance is uncommon, it appears occasionally, especially in the ipsilateral hippocampus. However, we found that concordance differentiates better in non‐HS cases, which does not completely align with findings that highlight concordance in HS cases.^6^, ^48^, ^52^ This could point to distinct etiologies. Although limited in frequency, concordant abnormalities when resected likely lead to ILAE_1,2_ outcomes and should be considered in surgical planning.

We analyzed GM and SWM abnormalities from a regional perspective, but it is crucial to acknowledge that epilepsy is a network disorder.^23^, ^53^, ^54^, ^55^ Surgical resection can significantly affect the epileptogenic network, potentially preventing further seizures even if regions deemed abnormal are not removed. Prior research demonstrated the impact of surgery on the structural connectome,^23^, ^55^ with certain regions being more critical due to their higher connectivity.

Balancing the removal and protection of SWM in epilepsy surgery requires careful consideration. SWM may play a role in the propagation of seizures and the localization of epileptogenic zones,^6^, ^11^, ^46^ but modern techniques, such as laser interstitial thermal therapy (LITT)^56^, ^57^ and historical approaches, such as amygdalohippocampectomy,^56^, ^58^ prioritize minimizing functional damage. To navigate this balance, a risk–benefit analysis is essential, where decisions are individualized to weigh seizure control against potential risks such as cognitive deficits.^59^, ^60^ Selective resection techniques, such as LITT, can be used to precisely target epileptogenic zones while sparing critical SWM.^56^, ^57^ Improved localization methods, supported by voxel‐based identification of SWM abnormalities,^20^ help refine SWM mapping. Finally, postoperative monitoring is crucial to assess SWM reorganization and functional recovery over time, guiding rehabilitation efforts accordingly.

This study has some limitations. We focused on TLE, whereas future studies could extend this to extra‐temporal cases.^6^, ^14^, ^47^, ^48^ In addition, only MD was included here, whereas other studies have also used fractional anisotropy, axial diffusivity, and radial diffusivity to assess WM abnormalities in epilepsy.^7^, ^13^, ^48^, ^61^ We selected MD as it reflects average diffusion in all directions, with higher MD values indicating more unrestricted diffusion, often linked to myelin disruption and increased extracellular space in WM,^38^, ^39^ which are common in epilepsy.^7^ Furthermore, we analyzed SWM only up to a 5 mm depth, while different depths could be used,^62^ and future studies could examine multiple depths to determine the most sensitive depth for SWM abnormalities. In addition, based on available histology, 80% of our HS cases were type I—the more common subtype associated with better surgical outcomes—which may introduce potential bias.^63^ Another limitation is that abnormalities were calculated against two small control cohorts. Although the replicability across the two cohorts gives confidence in our results, future research could benefit from normative diffusion‐weighted MRI models combined with T1‐weighted MRI models,^64^, ^65^, ^66^ trained on larger control samples, for a robust baseline. Finally, the variability in acquisition location and the interval between surgery and post‐operative MRI acquisition may represent a source of heterogeneity and should be considered as a potential limitation when interpreting the imaging‐based findings.

In summary, our study presents a method to investigate the relationship between GM and SWM abnormalities in TLE related to epilepsy surgery. We found that these abnormalities were present across the whole cohort and could provide vital insights for surgical decision‐making. Analyzing multiple types of abnormality (here GM and SWM) together substantially enhances differentiations of surgical outcomes, with an increased rate of living without disabling seizures when abnormal regions are concordant and resected. Our findings suggest that diffusion‐weighted MRI abnormalities complement traditional T1‐weighted scans, improving pre‐surgical assessments and seizure control, especially in complex cases, thus offering a more accurate prognosis for epilepsy.

## AUTHOR CONTRIBUTIONS

Peter N. Taylor: Writing – review and editing, supervision, conceptualization, and funding acquisition. Yujiang Wang: Resources and conceptualization. Andrew W. McEvoy: Resources. Gerard Hall: Resources and conceptualization. Callum Simpson: Resources. Jane de Tisi: Resources. John S Duncan: Resources, conceptualization, and writing – review and editing. Sjoerd B. Vos: Writing – review and editing, data curation, and resources. Gavin P. Winston: Writing – review and editing, investigation, data curation, resources, conceptualization, and funding acquisition. Csaba Kozma: Writing – review and editing, writing – original draft, visualization, methodology, formal analysis, data curation, and conceptualization. Jonathan Horsely: Supervision, conceptualization, and resources.

## FUNDING INFORMATION

CK: Csaba Kozma is supported by Epilepsy Research Institute UK: United Kingdom. P.N.T.: Peter NEal Taylor and Y.W.: Yujiang Wang are both supported by UKRI: United Kingdom Research and Innovation Future Leaders Fellowships (MR/T04294X/1, MR/V026569/1). G.P.W.: Gavin Paul Winston and scan acquisition was supported by the MRC: MEdical Research Council (G0802012, MR/M00841X/1). J.S.D: John S Duncan and J.d.T: JAne de Tisi are supported by the NIHR: National Institute of Health and Research UCLH/UCL: University College London Hospital/University College London Biomedical Research Centre.

## CONFLICT OF INTEREST STATEMENT

None of the authors has any conflict of interest to disclose. We confirm that we have read the Journal's position on issues involved in ethical publication and affirm that this report is consistent with those guidelines.

## Supporting information


Figure S1.

## Data Availability

Code and data to reproduce figures in the manuscript will be made available upon acceptance of the paper.
