# Supplementary material for: Combined impact of gray and superficial white matter abnormalities: Implications for epilepsy surgery
Source: Epilepsia. 2025 Jun 10;66(10):3688–99. doi: 10.1111/epi.18494 (PMC12605822; doi:10.1111/epi.18494)
Supplement: Supplementary file 1 — Figure S1. [file EPI-66-3688-s001.zip › epi18494-sup-0001-DataS1.docx]

# Supplementary

# S1 Discordance between GM and SWM abnormalities

**Figure S1: Proportion of patients with abnormality in either grey or superficial white matter region (discordance):** (A) Comparison of resected abnormal ROIs between ILAE${}_{1,2}$ and ILAE${}_{3+}$ patients using GM and SWM discordance. Each point represents a patient, with the darker line indicating the median.

The resection of ROIs containing either GM or SWM but not both (discordant) showed good differentiation of seizure outcomes better than individual modalities (AUC = 0.72, p $<$ 0.01; Figure S1). Discordant and union abnormalities were predominantly located in the ipsilateral hemisphere, particularly near the presumed epileptogenic zone (Figure S2).

# S2 Abnormality localization using the combination of GM and SWM: union, concordance, discordance

**Figure S2: Spatial distribution of combination of grey and superficial white matter abnormalities.** The proportion of individuals with abnormalities in each ROI for (A) union, (B) concordance and (C) discordance.

In the union approach, 35% of patients showed abnormalities in the ipsilateral temporal pole and 55% in the hippocampus, confirming the hippocampus as the most frequently affected region in the TLE cohort (Figure S2A). The union abnormalities were concentrated in the ipsilateral hemisphere, especially near the presumed epileptogenic zone (Figure S2A). Additionally, concordance between GM and SWM abnormalities occurred only 56% of cases, with the highest concordance observed in the ipsilateral hippocampus and adjacent SWM (15%; Figure S2B) and inferior temporal gyrus (3%). Finally, discordant GM and SWM abnormalities showed similar patterns as union abnormalities with 33% of patients showing abnormalities in the ipsilateral temporal pole and 39% in the hippocampus (Figure S2C). These results highlight the impactful contributions of GM and SWM, separately and together, underscoring the potential need for a multimodal approach to epileptic abnormalities.

# S3 Differentiate outcome in MRI negative patients

**Figure S3: Replication of surgical outcome findings in MRI negative individuals using GM, SWM and their combinations:** Comparison of resected abnormal ROIs between ILAE${}_{1,2}$ and ILAE${}_{3+}$ patients (A) using GM, (B) SWM, (C) union, (D) concordance, (E) and discordance. Each point represents a patient, with the darker line indicating the median.

We replicated our analysis, specifically, in MRI-negative individuals. Of the 143 individuals with TLE, only 20 were MRI-negative, limiting in-depth analysis for this subgroup. Despite the small sample size, here too, union and discordance approach effectively differentiated ILAE${}_{1,2}$ and ILAE${}_{3+}$ outcomes in MRI-negative cases (Union AUC = 0.81, p $<$ 0.05; Discordance AUC = 0.81, p $<$ 0.05; Figure S3). These promising findings warrant replication with a larger sample size.

# S4 Differentiate outcome separately in our two acquisition cohorts

**Figure S4: Replication of surgical outcome findings in two acquisition cohorts using GM, SWM and their combinations:** Comparison of resected abnormal ROIs in ILAE${}_{1,2}$ vs ILAE${}_{3+}$ patients using (A) old and (B) new imaging protocols across GM, SWM, union, concordance, and discordance metrics. Each point represents a patient, with a darker line marking the median.

Our data were collected using two acquisition protocols. The first cohort (87 patients, 29 controls) was scanned from 2009 to 2013 on a 3T GE Signa HDx scanner, with T1-weighted images (1.1 mm slices) and DWI using 52 directions (b = 1,200 s/mm²). The second cohort (56 patients, 67 controls) was scanned from 2014 to 2019 on a 3T GE MR750 scanner with improved gradients, T1-weighted images (1 mm slices), and DWI with 115 volumes across four b-values. Results show that in both cohorts, union and discordance best differentiated ILAE${}_{1,2}$ and ILAE${}_{3+}$ outcomes (Union old cohort AUC = 0.75, p $<$ 0.01; Discordance old cohort AUC = 0.75, p $<$ 0.01; Union new cohort AUC = 0.81, p $<$ 0.01; Discordance new cohort AUC = 0.82, p $<$ 0.01; Figure S4). The newer acquisition protocol, with improved diffusion quality, outperformed the older protocol, highlighting the importance of updated imaging standards for precise abnormality localization.

# S5 Abnormalities across parcellation schemes

**Figure S5: Replication of surgical outcome findings across parcellations:** Comparison of resected abnormal ROIs in ILAE${}_{1,2}$ and ILAE${}_{3+}$ patients in (A) 216 cortical + 14 subcortical and, (B) 114 cortical + 14 subcortical regions across GM, SWM, union, concordance, and discordance metrics. Each point represents a patient, with a darker line marking the median.

We used a 446-region parcellation in the main manuscript. Here, we show consistent results across alternative parcellations (Figure S5). Our results show that union and discordance best differentiate between ILAE${}_{1,2}$ and ILAE${}_{3+}$ cases across all parcellations.

# S6 Abnormalities in HS vs non-HS

**Figure S6: Replication of surgical outcome findings in HS and non-HS patients using GM, SWM and their combinations:** Comparison of resected abnormal ROIs in ILAE${}_{1,2}$ vs ILAE${}_{3+}$ patients splitting to (A) HS and (B) non-HS cases across GM, SWM, union, concordance, and discordance metrics. Each point represents a patient, with a darker line marking the median.

The results held for both HS and non-HS cases, except for GM-only abnormalities (HS AUC = 0.68, p = 0.02; non-HS AUC = 0.59, p = 0.16). GM, SWM, and their combinations better differentiated ILAE${}_{1,2}$ and ILAE${}_{3+}$ outcomes in HS cases (Figure S6). Surprisingly, concordance differentiated outcomes more effectively in non-HS cases (HS AUC = 0.62, p = 0.34; non-HS AUC = 0.77, p = 0.07), though caution is needed due to the small number of concordant cases.

# S7 AUCs for distinguishing ILAE1,2 and ILAE3+ in Years 2–5.

**Figure S7: Replication of surgical outcome findings across ILAE outcomes Year 2–5:** Comparison of resected abnormal ROIs in ILAE${}_{1,2}$ and ILAE${}_{3+}$ patients in (A) ILAE Year 2, (B) ILAE Year 3, (C) ILAE Year 4, (D) ILAE Year 5 across GM, SWM, union, concordance, and discordance metrics.

Postoperatively, we looked at seizure outcomes at 12 months in the main manuscript, while here we present the results for Year 2–5 (Figure S7).

# S8 Comparison of GM and SWM z-score correlation: Patients vs. Controls

**Figure S8: Distributions of correlations across GM and SWM z-scores:** Comparison of correlations across modalities for patients and controls.

The correlation values between GM and SWM z-scores in patients did not significantly differ from those in controls (W = 7119, p = 0.6592) (Figure S8). This potentially means that the two modalities present complementary abnormalities that are contributing to epileptogenic activity.

# S9 Bootstrapping analysis on the individual ROC analyses

**Figure S9: Bootstrapping analysis on the individual ROC curves:** Distribution of AUC values based on the resampled datasets for GM, SWM, Union of GM and SWM, Concordance and Discordance across GM and SWM. Each dot is an individual AUC value based on the corresponding resampled dataset.

We conducted a bootstrapping analysis on the individual ROC curves, using 1,000 bootstrap samples with replacement, each maintaining the original sample size. This approach allowed us to assess the robustness of the AUC values in distinguishing between outcome groups. The results demonstrated that the AUCs remained consistent and stable across the resampled datasets, indicating the reliability of the model’s performance (Figure S9.1).

# Table S10 Average proportion of abnormal regions and the proportion of resected abnormal regions

|  | GM | WM | Union | Concordance | Discordance |
| --- | --- | --- | --- | --- | --- |
| Mean proportion of regions identified as abnormal | 1.5% | 3.8% | 5.1% | 0.2% | 4.8% |
| Mean proportion of abnormal regions resected | 17% | 18.2% | 16.5% | 33.8% | 15.7% |

# Table S11 Definition of terms

| Term | Definition |
| --- | --- |
| Union | The union of two modalities (Grey Matter and Superficial White Matter) is the set that contains all the regions of interest that are deemed abnormal either in Grey Matter or Superficial White Matter, or both. |
| Concordance | The concordance of two modalities (Grey Matter and Superficial White Matter) is the set that contains all the regions of interest that are deemed abnormal in both modalities. In set theory this is also known as the intersection. |
| Discordance | The discordance of two modalities (Grey Matter and Superficial White Matter) is the set that contains all the regions of interest that are deemed abnormal either in Grey Matter or Superficial White Matter, but not in both. |

# S12 Average resection overlap for all individuals

**Figure S12 Mean resection overlap across all patients:** Distribution of resection overlap across all patients for ipsilateral and contralateral hemispheres.

All resections in this TLE cohort involved the ipsilateral temporal lobe, specifically the anterior sections of the superior, middle, and inferior temporal gyri, the temporal pole, fusiform, and entorhinal regions, as well as the ipsilateral hippocampus and amygdala subcortically.
