# Supplementary figures and images for: Combined impact of gray and superficial white matter abnormalities: Implications for epilepsy surgery
Source: Epilepsia. 2025 Jun 10;66(10):3688–99. doi: 10.1111/epi.18494 (PMC12605822; doi:10.1111/epi.18494)

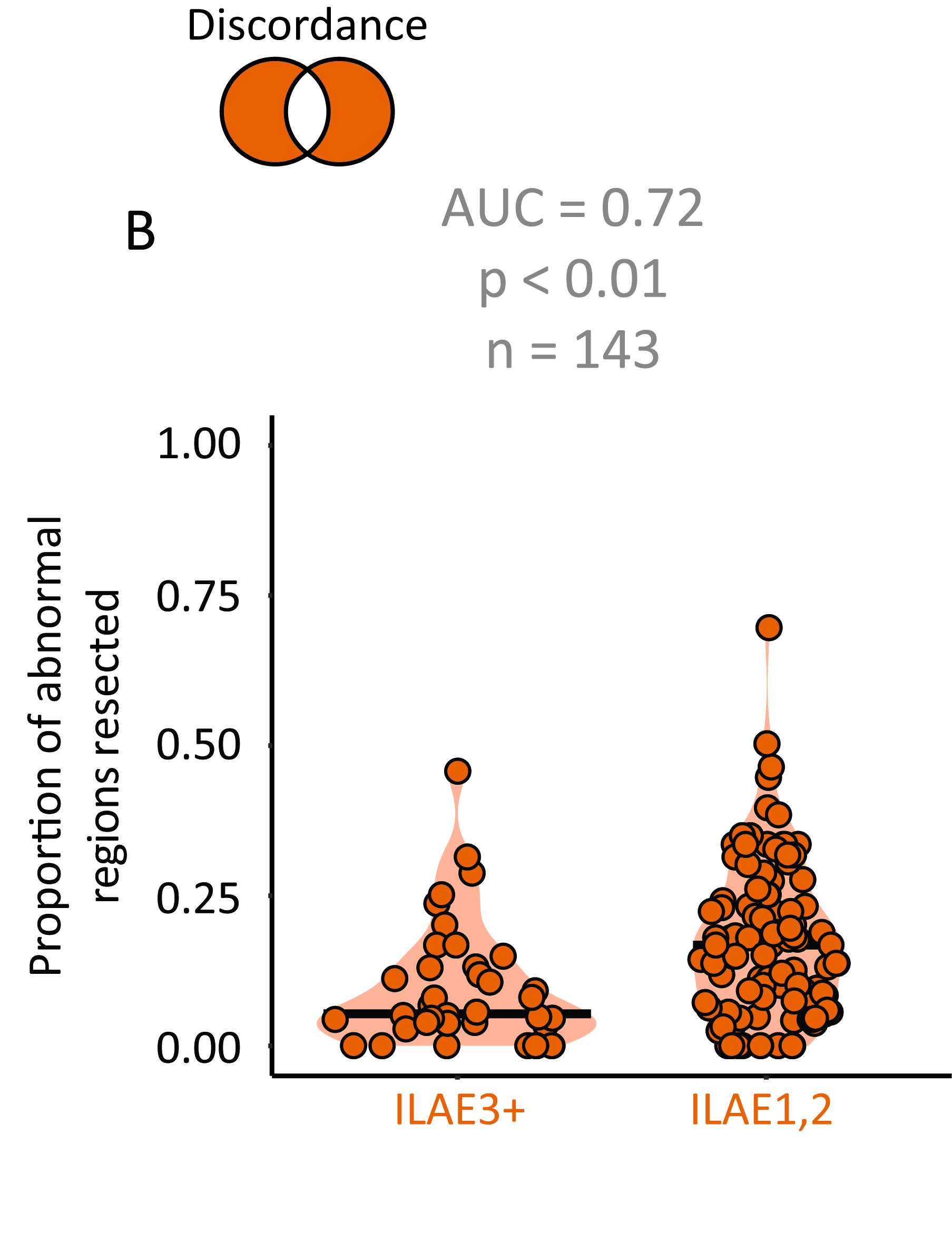

Supplement: Supplementary file 1 — Figure S1. [file EPI-66-3688-s001.zip › epi18494-sup-0002-Supinfo1@Supplementary_1.tif]

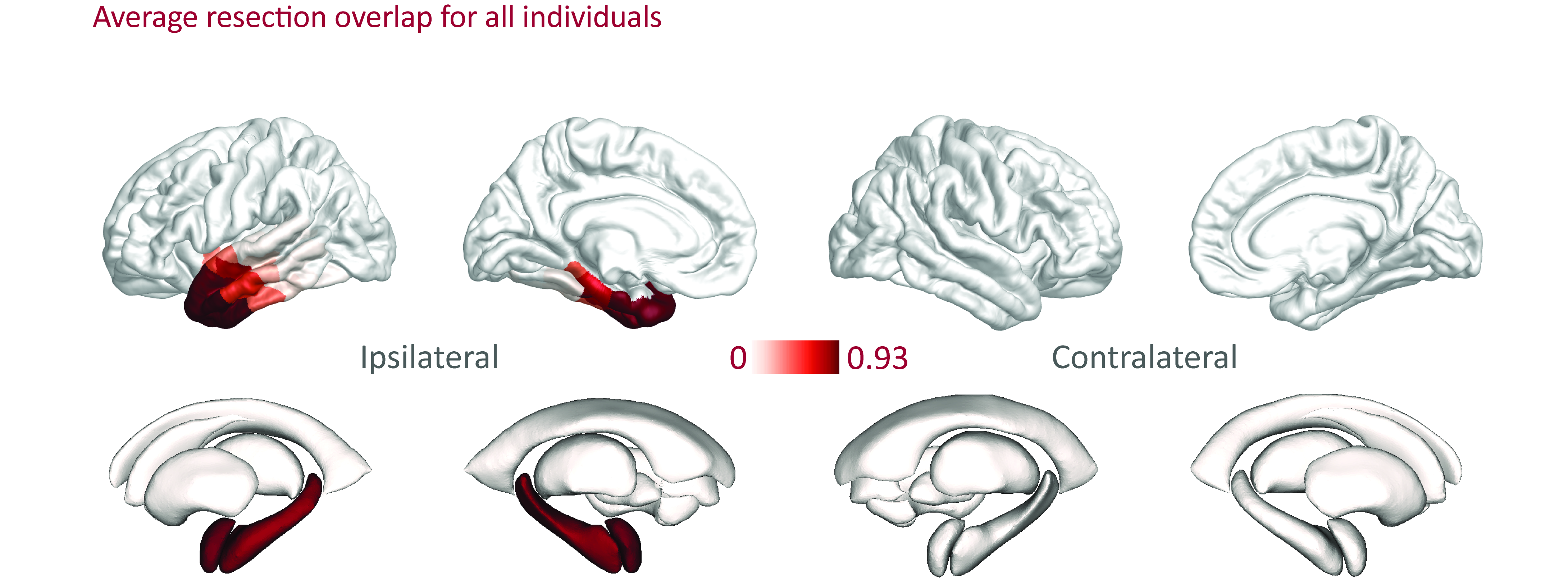

Supplement: Supplementary file 1 — Figure S1. [file EPI-66-3688-s001.zip › epi18494-sup-0003-Supinfo2@Supplementary_12.tif]

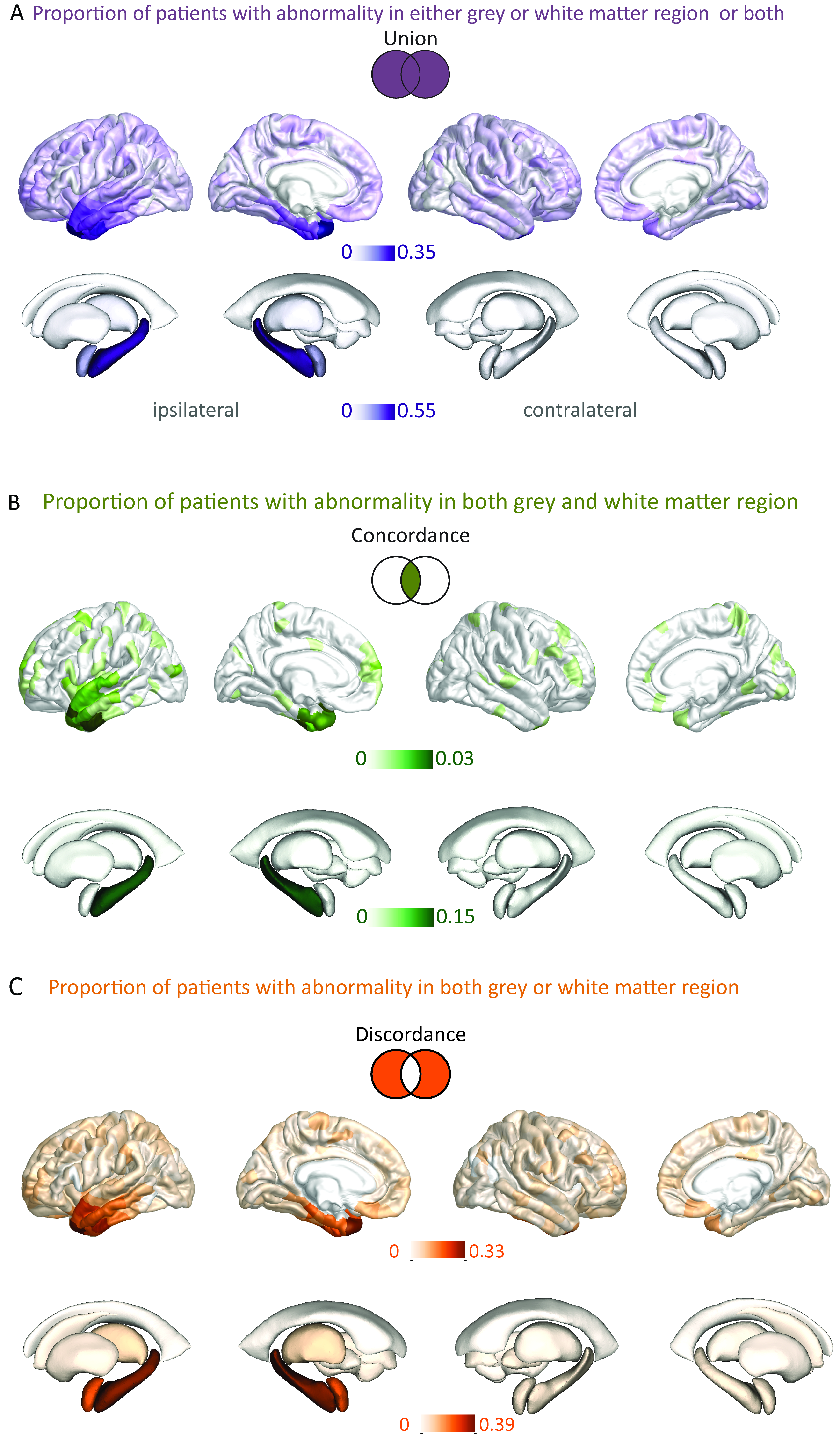

Supplement: Supplementary file 1 — Figure S1. [file EPI-66-3688-s001.zip › epi18494-sup-0004-Supinfo3@Supplementary_2.tif]

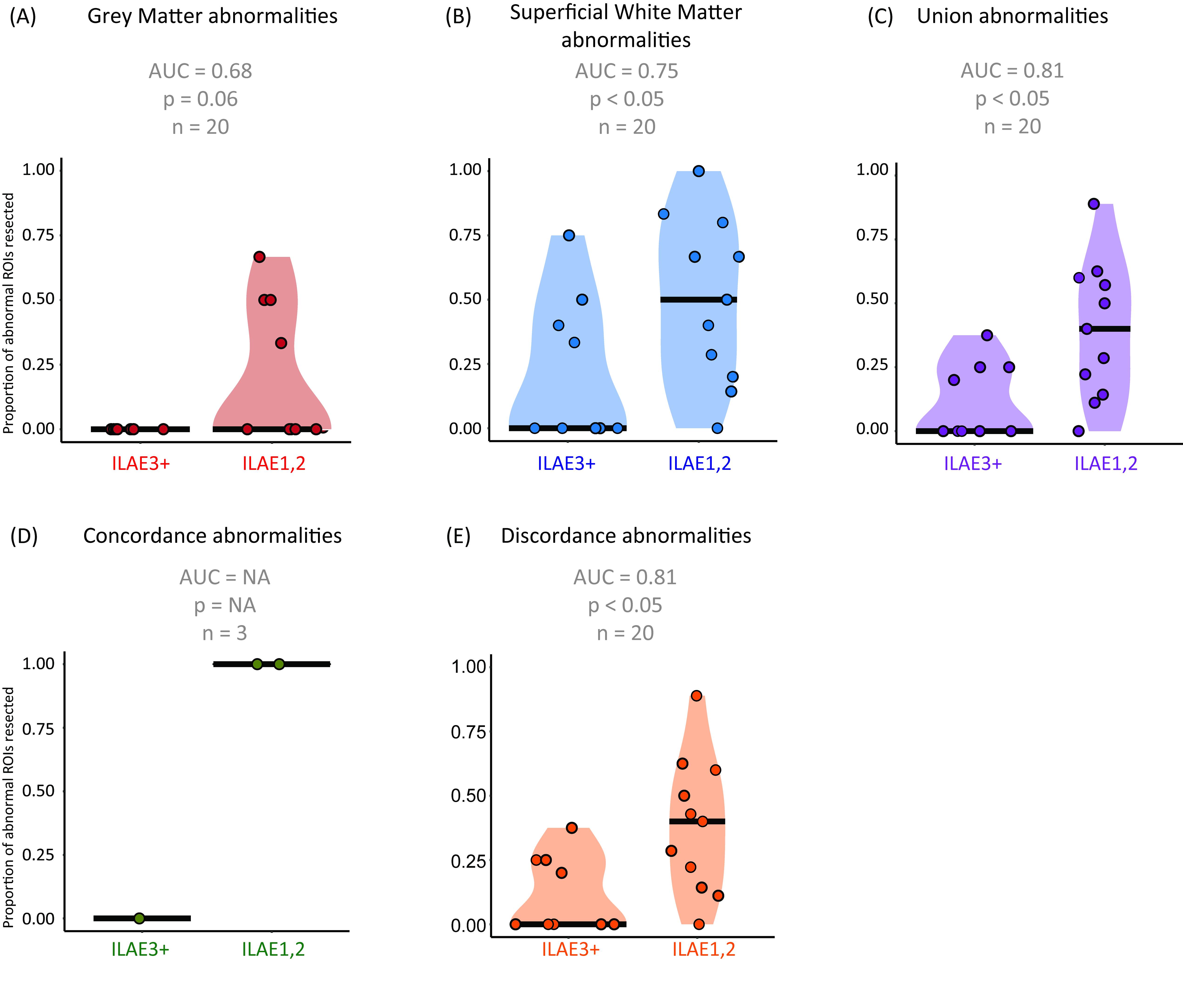

Supplement: Supplementary file 1 — Figure S1. [file EPI-66-3688-s001.zip › epi18494-sup-0005-Supinfo4@Supplementary_3.tif]

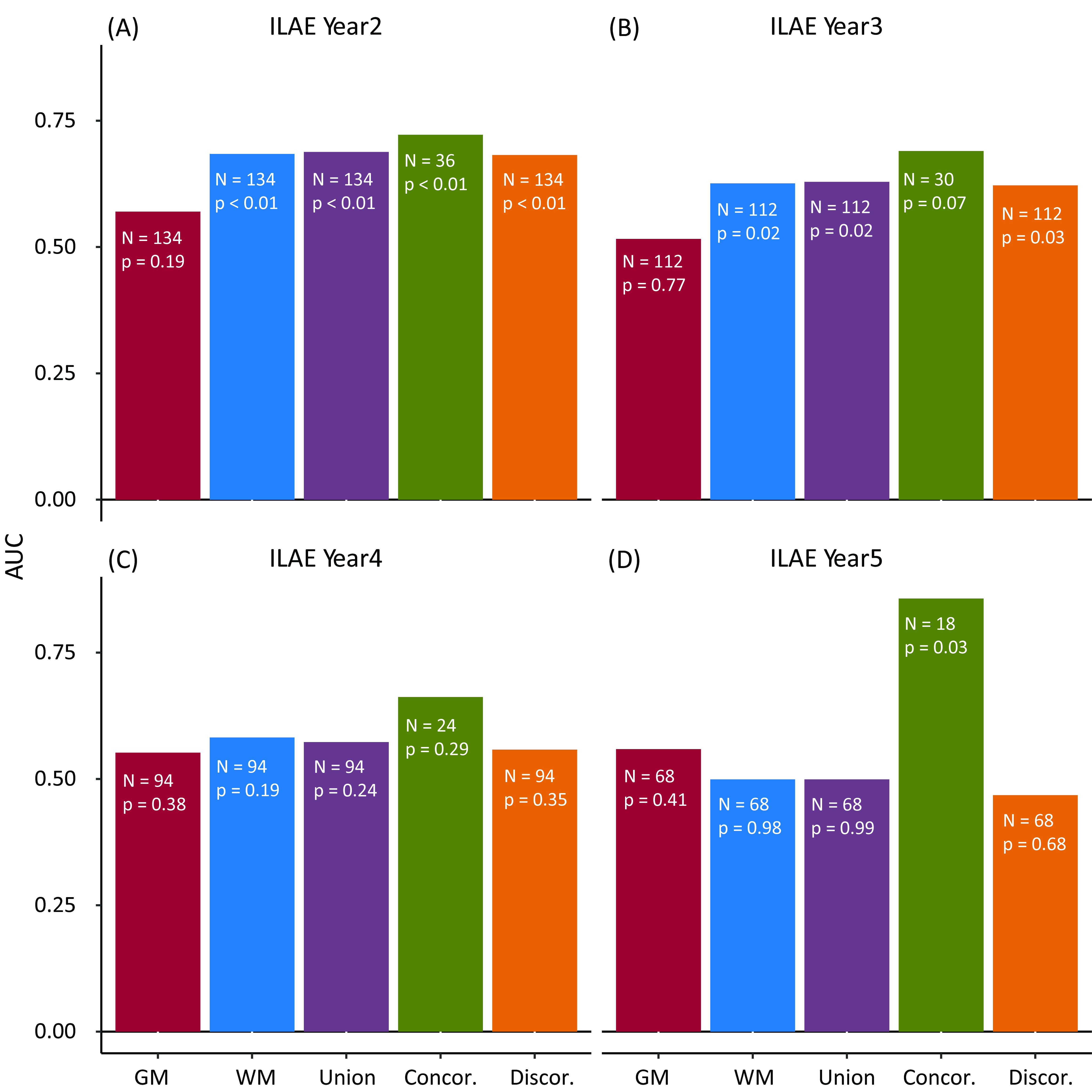

Supplement: Supplementary file 1 — Figure S1. [file EPI-66-3688-s001.zip › epi18494-sup-0009-Supinfo8@Supplementary_7.tif]

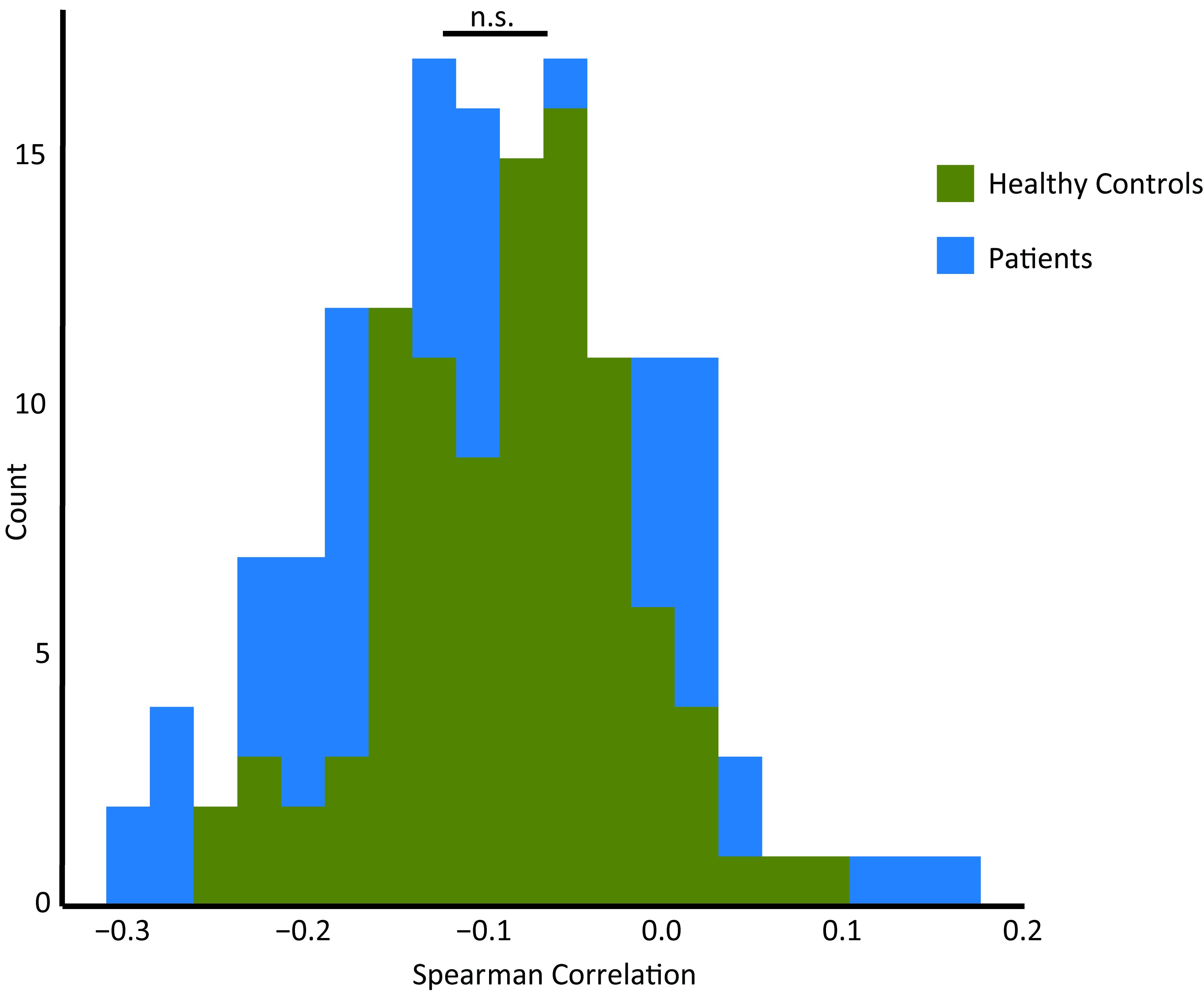

Supplement: Supplementary file 1 — Figure S1. [file EPI-66-3688-s001.zip › epi18494-sup-0010-Supinfo9@Supplementary_8.tif]

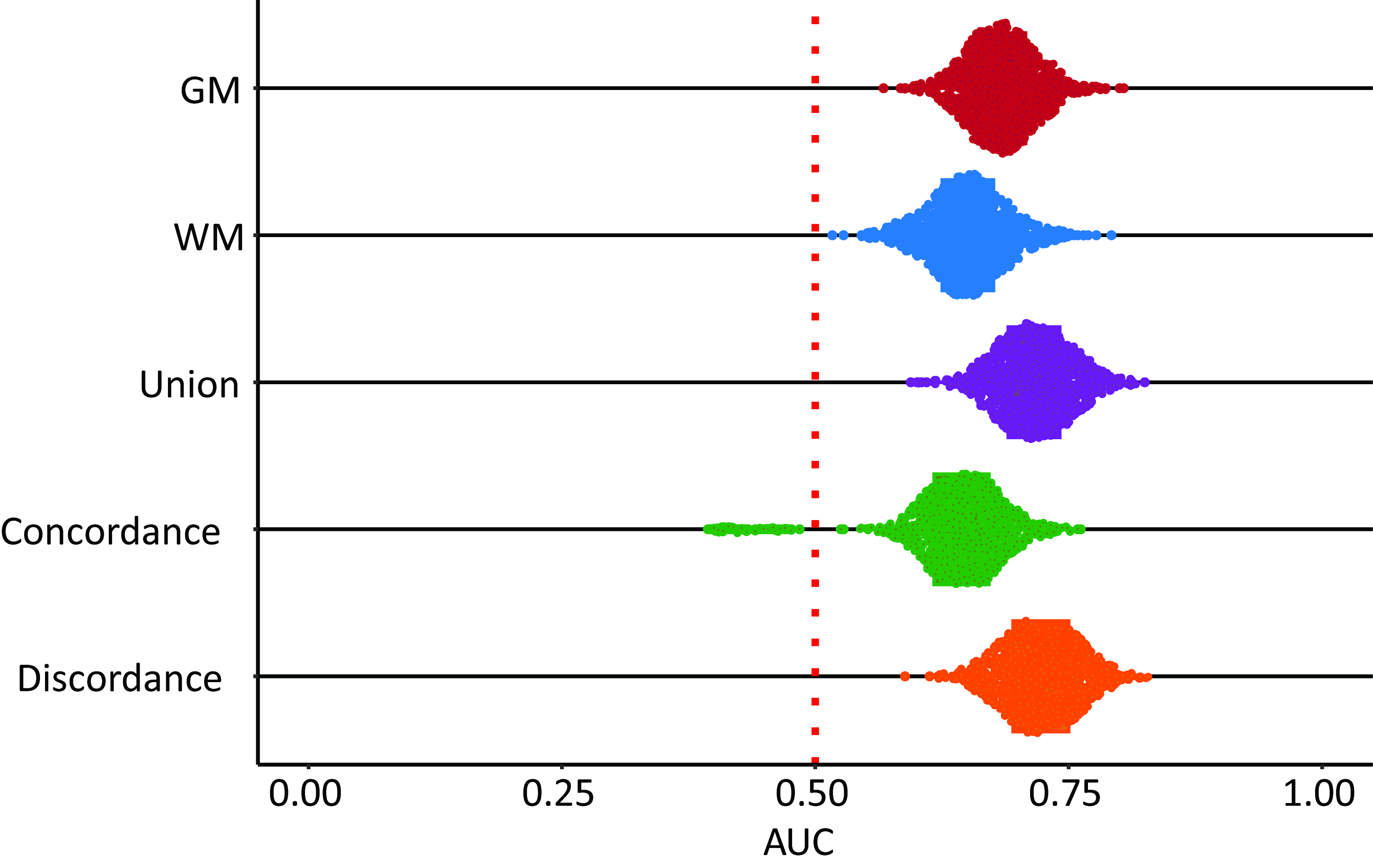

Supplement: Supplementary file 1 — Figure S1. [file EPI-66-3688-s001.zip › epi18494-sup-0011-Supinfo10@Supplementary_9.tif]
